# The first dataset of vascular plant species occurrences on kurgans in Southern Ukraine

**DOI:** 10.3897/BDJ.10.e96879

**Published:** 2023-01-03

**Authors:** Ivan Moysiyenko, Barbara Sudnik-Wójcikowska, Iwona Dembicz, Maria Zachwatowicz, Nadiia Skobel

**Affiliations:** 1 Kherson State University, Faculty of Biology, Geography and Ecology, Kherson, Ukraine Kherson State University, Faculty of Biology, Geography and Ecology Kherson Ukraine; 2 University of Warsaw, Faculty of Biology and Biological and Chemical Research Centre, Warsaw, Poland University of Warsaw, Faculty of Biology and Biological and Chemical Research Centre Warsaw Poland; 3 University of Warsaw, Faculty of Geography and Regional Studies, Warsaw, Poland University of Warsaw, Faculty of Geography and Regional Studies Warsaw Poland

**Keywords:** Kurgans' flora, barrows, refugia of steppe flora, floristic diversity, desert steppe, grass steppe, herb-rich grass steppe, forest steppe, protection of kurgans, Kherson, Mykolaiv, Poltava, Cherkasy, Kirovograd regions, Ukraine

## Abstract

**Background:**

The dataset contains the records of vascular plant species occurrences and distribution on Ukrainian kurgans (burial mounds, barrows), located in various zones of steppe vegetation: desert steppe, grass steppe, herb-rich grass steppe and forest steppe. Much of the studied kurgans belongs to the territory historically known as the “Wild Fields”. Besides the occurrence data, the publication presents a comparison of the floristic richness amongst five microhabitats distinguished on kurgans (top, northern slope, northern bottom, southern slope, and southern bottom) and amongst kurgans located in different steppe zones. The Original publication includes 721 species of vascular plants) within four vegetation zone (desert steppe, grass steppe, herb-rich grass steppe and forest steppe). The report shows also sozological value of kurgans in southern Ukraine, as they play a role of steppe habitat islands in a landscape almost completely transformed to arable land. The obtained flora inventory was analyzed in various aspects. This occurrence dataset is the first public record of species from kurgans in Ukraine.

**New information:**

This is the first occurrence dataset from kurgans in Ukraine. The dataset includes 28,456 occurrences of vascular plants recorded in the years 2004-2009 on Ukrainian kurgans. The dataset includes information about 1446 occurrences of rare species on kurgans (69 species). It contains information on the kurgan flora within four vegetation zone (desert steppe, grass steppe, herb-rich grass steppe and forest steppe) on the area ca. 32000 km^2^. Of the approximately 450 mounds visited, the ones with the best preserved vegetation cover were selected. For each of 106 investigated mounds, floristic lists from five microhabitats were compiled - 530 lists in total.

## Introduction

A "kurgan" is a word derived from the old Turkish language meaning: refuge, fortress, but also high grave. A kurgan is defined as a mound of earth (and/or pile of stones), often conical or hemispherical in shape, constructed over a burial chamber, containing a single or multiple graves. The following terms are used as synonyms: barrow, burial mound, tumulus or tomb.

Kurgans are not associated with specific climatic-vegetation zones, but most of them are located in the steppes. Barrows were built by nomadic peoples from the Eurasian steppes, but also by Indian tribes from the prairies of North America. Kurgans in Eurasia were built from the Eneolithic through the Bronze and Iron Ages, up to the early Middle Ages and provide evidence of migrations and wars conducted by nomadic or semi-sedentary peoples. Most of the barrows were attributed to: Cimmerians, Scythians, Sarmatians (Iron Age) and later: Huns, Bulgarians, Magyars, Polovtsians, Nogays and others. The earliest information about the barrows on the north shore of the Black Sea was provided by Herodotus in "The Histories". Some sporadic information also comes from the late Middle Ages (Sudnik-Wójcikowska et al. 2012).

The archaeological value of kurgans has been widely recognised. However, less is known about the floristic value of these man-made structures. It is surprising that, by the end of the 20^th^ century, the specific flora and fauna of barrows was the subject of only a few studies in central and south-eastern Europe (Deák et al. 2016a, Deák et al. 2016b). Investigations were carried, for example, in Bulgaria (Paczoski 1933) and Hungary (Ecsedy 1979). However, since the beginning of the 21^st^ century, interest in this topic has been growing and there are more and more publications devoted to these issues: in Bulgaria (e.g. Apostolova et al. (2020)), Hungary (Barczi 2003, Barczi et al. 2004, Bede et al. 2019, Deák et al. 2016a, Deák et al. 2019, Penksza and Joó 2002), in Kazakhstan (e.g. Deák et al. (2018)), in Poland (e.g. Cwener and Towpasz (2003), Cwener (2004), Cwener (2005), Towpasz (2006)), in Russia (e.g.Bykov and Khrustaleva 2008, Dzybov 2006) and in Ukraine (e.g. Boreiko et al. 2002, Bozhko 2008, Fisun 2008, Lystopad 2009, [Bibr B8173774][Bibr B8173774], Boreiko et al. (2002), Bozhko (2008), Fisun (2008), Lystopad (2009)),as well as newer publications from the authors of this database (see the section: Sampling description).

The steppe biome is considered to be the most transformed of all biomes in the world. It is estimated that 82–90% of steppe vegetation in Ukraine has been destroyed due to agricultural practices and the development of human settlements. In consequence, its area has been reduced 50-fold during the past 2000 years. Kurgans constituted an impediment to large-scale agriculture. Before the “taming of the steppe”, which occurred about 200 years ago (e.g. Sunderland (2004), Khodarkovsky (2009)), the barrows in southern Ukraine were surrounded by virgin steppe vegetation, which promoted formation of the plant cover similar to the natural steppe vegetation. Kurgans are some of the most characteristic features of the Ukrainian landscape. Ukraine is referred to as "the land of kurgans" and it is hard to imagine the history and landscape of Ukraine without the barrows. The original number of kurgans in Ukraine is estimated at half a million, of which probably about 100,000 (according to other authors, 50,000 or 150,000) survive to this day. A high number of barrows were destroyed because they hindered farming activities. Those that survived (usually larger ones) became isolated because they were surrounded by extensive arable fields. The larger barrows that remained, vary in terms of the state of preservation of their plant cover. Nevertheless, they resemble the “native flora islands” in ‘‘the ocean’’ of arable fields. The best preserved kurgans are of high conservation value and can play a significant role as refugia of the steppe flora (Sudnik-Wójcikowska et al. 2011, Sudnik-Wójcikowska et al. 2012).

Our field investigation (2004-2009) has shown the great importance of kurgans in preserving biodiversity. The examined barrows were spread over an area of about 32,000 km^2^ in Kherson, Mykolaiv, Poltava, Cherkasy and Kirovograd regions. Of the approximately 450 visited kurgans, we chose 106 with the most interesting flora. The kurgan flora database includes 719 taxa of vascular plants. The total number of occurrences is 28,456. Amongst the recorded taxa, the notable number is protected and listed in the Red Data Book of Plants of Ukraine (Ed. Didukh 2009) and other levels of protection. The burial mounds should, therefore, be particularly protected, not only as the monuments of archaeology, but also because of their natural value.

## General description

### Purpose

Barrows as the objects of value of material culture were recognised by archaeologists quite early, while much less attention was paid to their importance as natural sites. It is surprising how few publications on this subject appeared until the end of the 20^th^ century (one of the few: Paczoski (1914)). In recent years, there has been a growing interest in kurgans. Natural scientists are looking for evidence to protect them. Our long-term floristic survey serves this purpose.

The floristic data, collected on the kurgans, were compiled into a database. We used the database to achieve the following goals:

1. to characterise the total flora of the barrows;
2. to compare the flora of microhabitats within the kurgans;
3. to present the similarity of flora of the kurgans and the flora of the climatic-vegetation zones in which the barrows are located;
4. to indicate the most valuable species (legally protected or listed in the Red Data Book of Plants of Ukraine (Ed. Didukh (2009));
5. to emphasise the role of kurgans in the process of restitution of the steppes - the most damaged biome in the world.

## Project description

### Personnel

Ivan Moysiyenko, Barbara Sudnik-Wójcikowska

### Funding

The collecting of floristic data, field investigations and further data analysis were supported by the projects of the Polish Ministry of Science and Higher Education: "Kurgans as a refuge of the steppe flora in the agricultural landscape of southern Ukraine” 2 PO4G 046 27 (2004-2007) and "Kurgans as centers of floristic diversity requiring special protection in the anthropogenic landscape in the zone of steppes and forest steppe of southern Ukraine” NN 304 081835 (2008-2011). The data publishing was supported by the project: “Impact of war on cultural heritage sites as refugia of biological diversity in Ukraine” D596 (2022).

The dataset was prepared in collaboration with the University of Warsaw (Contract of employment BSP-NN-10757/22).

We are also grateful to "Finnish Biodiversity Information Facility (FinBIF)" for their call for authors in the project “Northern Eurasia 2022”.

## Sampling methods

### Study extent

**Figure 1.**
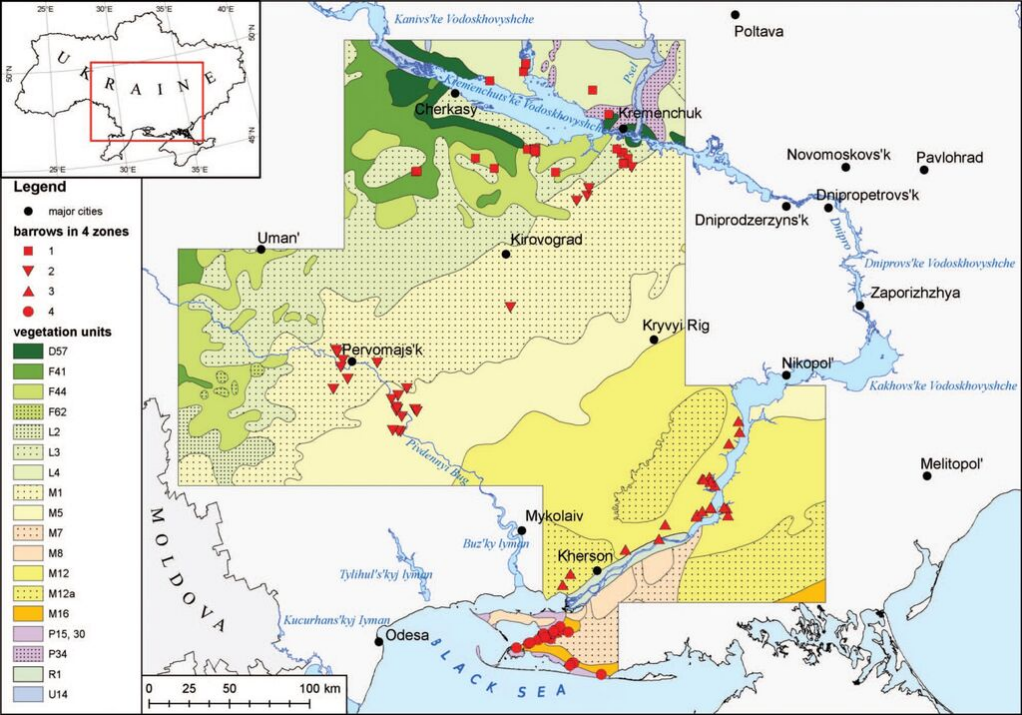
Distribution of the kurgans investigated in the steppe and forest steppe zones in southern Ukraine: (1) square – kurgans in the forest-steppe zone; (2) upside-down triangle – kurgans in the west and central Pontic herb-grass steppe and west and central Pontic herb-rich grass steppe zone; (3) triangle – kurgans in the west Pontic grass steppe zone; (4) circle – kurgans in the desert steppe zone. Designations according to Bohn et al. (2000): The forest steppe zone: D57 – southeast European herb- and grass-rich xerophytic pine and oak pine forests, F41 – east Polish- Ukrainian lime-pedunculate oak-hornbeam forests, F44 – Podolian-Moldavian thermophilous hornbeam-pedunculate oak forests; F62 – east pre-Carpathian-Moldavian sessile oak-hornbeam forests; L2 – Vohlyn-Podolian meadow steppes; L3 – Moldavian-Ukrainian meadow steppes; L4 – south Sarmatian meadow steppes; the steppe zones: M1 – west and central Pontic herb-rich grass steppes; M5 – west and central Pontic herb-grass steppes; M7 – Pontic hemi-psammophytic herb grass steppes; M8 – Pontic psammophytic herb grass steppes; M12 – west Pontic grass steppes; M12a – west Pontic grass steppes in combination with halophyte vegetation (solonchak); M16 – west and central Pontic desert steppes in combination with halophyte vegetation (solonchak, solonetz); P15 – west and central Pontic sand-dune vegetation; P30 – west Pontic halophytic vegetation; P34 – west and east Pontic salt meadows; R1 – freshwater tall reed swamps; U14 – Pontic hardwood alluvial forests (Sudnik-Wójcikowska et al. 2012).

The study area is located within four climatic-vegetation zones (three zones of the steppe and the forest steppe zone). Administratively, the area is located in the following regions of Ukraine: Kherson, Mykolaiv, Poltava, Cherkasy and Kirovograd (Fig. 1).

The examined barrows were spread over an area of approximately 32000 km^2^. Historically, most of the studied area is referred to as a "Wild Field".

**Figure 2.**
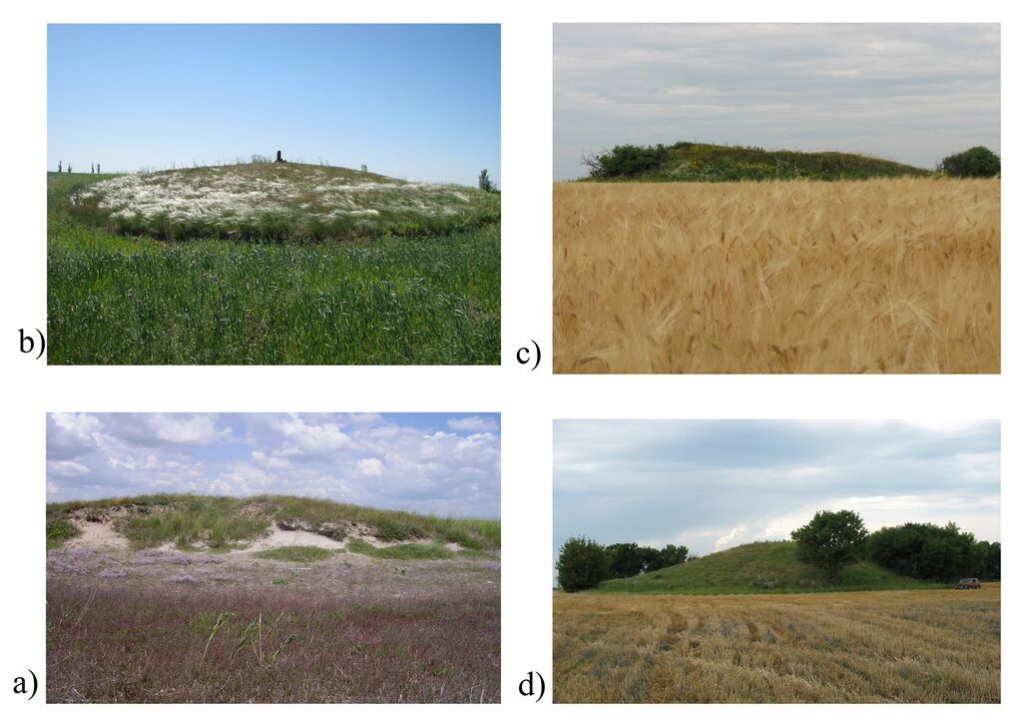
Kurgans in different zones: **a)** the desert steppe; **b)** the grass steppe; **c)** the rich-grass steppe; **d)** the forest steppe (photo Ivan Moysiyenko).

The field investigations of the kurgan’s flora were carried out for 6 years (at least 2-3 times per year; ca. 6-8 weeks per year), during the growing seasons of 2004-2009, successively in each of the steppe and forest-steppe zones (Fig. 2, Table 1)

a) the west and central Pontic desert steppe (= desert steppe) zone: 2004- 2006;

b) the west Pontic grass steppe (= grass steppe or proper steppe) zone: 2004-2006;

c) the west and central Pontic herb-rich grass steppe (= the herb-rich grass steppe) zone: 2006-2009;

d) the forest steppe zone: 2006-2009.

### Sampling description

We assumed that the flora of 25-29 well-preserved kurgans would be representative of each of the distinguished zones. The barrows were more or less evenly distributed in the zones of steppe and forest steppe. From amongst 450 visited kurgans, 106 of the most floristically valuable were selected. The selected mounds had to meet certain criteria: (1) height over 3 m, (2) relatively good state of preservation and (3) the presence of steppe vegetation and flora, especially tufted grasses from the genera *Stipa*, *Festuca*, *Koeleria* and *Bothriochloa* (further north).

For each kurgan, the identification number was provided, depending on the location:

a) the desert steppe zone: D1-D26;

b) the grass steppe zone: P1-P26;

c) the herb-rich grass steppe zone: R1-R29;

d) the forest steppe zone: F1-F25.

A complete inventory of the flora of vascular plants was carried out on the selected mounds. Every kurgan was examined at least 2-3 times during the growing season (spring, summer and autumn). Each of the 106 examined kurgans was divided into five microhabitats (T – the top of the barrow, Ss – the southern slope, Sn – the northern slope, Bs – the southern foot, Bn – the northern foot, see Floristic richness of microhabitats on kurgans). We determined the abundance of individual species in each of the microhabitats according to a simple 3-point scale (rare, fairly frequent, common species).

A separate floristic list was prepared for each microhabitat. We filled out special forms for the flora inventory. The identification of vascular plant species was held in the field. Specimens that could not be identified in the field were collected to the Kherson State University Laboratory of Plant Ecology and Environmental Protection.

Finally, the collective list of kurgans’ flora includes 721 taxa (Moysiyenko and Sudnik-Wójcikowska 2006a, Moysiyenko and Sudnik-Wójcikowska 2006b, Moysiyenko and Sudnik-Wójcikowska 2009, Moysiyenko and Sudnik-Wójcikowska 2010, Sudnik-Wójcikowska and Moysiyenko 2006, Sudnik-Wójcikowska and Moysiyenko 2010). The lists were the subject to further analysis (Moysiyenko and Sudnik-Wójcikowska 2008a, Moysiyenko and Sudnik-Wójcikowska 2008b, Moysiyenko and Sudnik-Wójcikowska 2010, Moysiyenko et al. 2014, Moysiyenko et al. 2022, Sudnik-Wójcikowska and Moysiyenko 2008a, Sudnik-Wójcikowska and Moysiyenko 2008b, Sudnik-Wójcikowska and Moysiyenko 2011a, Sudnik-Wójcikowska and Moysiyenko 2011b, Sudnik-Wójcikowska et al. 2012, Sudnik-Wójcikowska et al. 2011).

The documentation, in the form of herbarium sheets, has been deposited in the herbaria of the Herbarium of Kherson State University (more than 400 herbarium sheets in KHER) and the Herbarium of the Faculty of Biology of the University of Warsaw (about 200 herbarium sheets in WA).

### Quality control

The collected materials were verified in the Herbarium of Kherson State University (KHER), Herbarium of the Faculty of Biology of the University of Warsaw (WA) and herbarium in the Paczoski Museum of Kherson. Identification of some specimens was consulted with botanists from the M.G. Kholodny Institute of Botany, National Academy of Sciences of Ukraine. Species identification extracted from peer-reviewed scientific publication were taken as is, but checked for name misspelling against GBIF Species Matching tool.

Coordinates of records were checked using Google Earth service (Google Earth 2022).

### Step description

The following steps were taken:

1. The study of vascular plant flora on kurgans in southern Ukraine was carried out for 6 years (2004-2009). We conducted the research in four climatic vegetation zones, starting from the south (Black Sea coast), gradually moving towards the north (central Ukraine):

a) the desert steppe zone: 2004- 2006 (26 kurgans);

b) the grass steppe: 2004-2006 (26 kurgans);

c) the herb-rich grass steppe: 2006-2009 (29 kurgans);

d) the forest steppe: 2006-2009 (25 kurgans).

2. Each of the 106 examined kurgans was divided into five microhabitats (the top of the barrow, northern and southern slopes, the southern and northern foots). For each microhabitat and barrow, we prepared a floristic list (530 in total). We determined the abundance of individual species in each of the microhabitats according to a simple 3-point scale.

3. To make the lists of flora comparable, we strived to visit each kurgan at different times of the growing season (spring, summer, autumn). Thus, the floristic lists were successively supplemented.

4. We collected herbarium documentation (a total of about 600 sheets deposited in two university herbaria (KHER - Kherson State University and WA - University of Warsaw) and photographic documentation.

5. The obtained census of the kurgan flora includes 719 species and 28,456 occurrences compiled in an .CSV file.

6. Data were post-processed using Darwin Core terms (Wieczorek et al. 2012).

7. Data managment and cleaning was performed using OpenRefine (OpenRefine 2022).

## Geographic coverage

### Description

The study area is located in the Black Sea Lowland and Dnieper Upland, within the Kherson, Mykolaiv, Kirovograd, Cherkasy and Poltava regions, in four climatic-vegetation zones (Fig. 1). It covers a large area of southern and central Ukraine (about 32,000 km^2^). The concentration of kurgans in this area is greater than anywhere else in Europe, although they vary in their origin, history, degree of isolation and the intensity of anthropogenic factors. The area is diverse in terms of climate, soil and history of use. The amount of rainfall gradually increases from south to north and the annual temperatures decrease. Hence, the characteristics of the steppes change in the following zones (Bohn et al. 2000):

- The desert steppe. The steppe occupies a narrow strip along the coast of the Black and Azov seas. The mean precipitation here does not exceed 300 mm per year. Chestnut soils predominate in the complex with solonchaks. Most of the vegetation is dominated by clump grasses (*Stipa*, *Festuca*, *Agropyron*), numerous species of the genus *Artemisia* and halophytes (mainly Amaranthaceae: *Camphorosma*, *Salicornia*, *Bassia*, *Suaeda*, *Salsola*). Due to its salinity, the area is partly closed for use or used mainly as pastures.

- The grass steppe - located to the north of the desert steppe. Together with the next zone, it covers a strip 50 to 300 km wide. The soils are fertile. Dark chestnut soils predominate, passing northwards in southern chernozems with a thick layer of humus. Average annual rainfall ranges from 300 mm in the south to 400 mm in the north. Clump grasses of the genera *Stipa*, *Festuca* and *Koeleria* dominate, yet the area is heavily transformed by agricultural use.

- The herb-rich grass steppe - with increasing rainfall (up to 450 mm per year) and soil fertility, the share of dicotyledonous perennials and creeping grasses is growing. The area is intensively used for agriculture (ploughed even to 95%) and the steppe has survived only in marginal areas, the least useful for agriculture, for example, the balkas, the river valleys, of the outcrops of granite or limestone.

- The forest steppe – reaches the furthest north, where the mean annual rainfall is 450–750 mm. The humidity is only slightly lower than in the forest zone. On fertile soils (black earths, rendzinas, grey forest soils), a macromosaic of forests (mainly thermophilic deciduous) and very rich in species flowering (meadow) steppe developed. The forest steppe is very intensively transformed by agriculture.

### Coordinates

46.134 and 50.205 Latitude; 26.851 and 38.32 Longitude.

## Taxonomic coverage

### Description

Designation to which ranks of taxa the finds belong: most are identified by species, genus, presence of subspecies, forms.

According to GBIF Backbone Taxonomy (GBIF Secretariat 2021), our dataset include 719 taxa of vascular flora. Three taxa have been identified only to genus, 711 to species and five to subspecies. The original publication (Sudnik-Wójcikowska et al. 2012) includes 721 species of vascular plants, according to the checklist of vascular plants of Ukraine (Mosyakin and Fedoronchuk 1999). The dominant families Asteraceae, Fabaceae are important in steppe habitats as they are attractive to pollinators such as Lepidoptera, wild *Apis* and other Diptera. The dataset includes 30 orders (Apiales, Asparagales, Asterales, Boraginales, Brassicales, Caryophyllales, Celastrales, Cornales, Dipsacales, Ephedrales, Ericales, Fabales, Fagales, Gentianales, Geraniales, Lamiales, Liliales, Malpighiales, Malvales, Myrtales, Oxalidales, Piperales, Poales, Ranunculales, Rosales, Santalales, Sapindales, Saxifragales, Solanales and Zygophyllales) and 69 families (Adoxaceae, Amaranthaceae, Amaryllidaceae, Anacardiaceae, Apiaceae, Apocynaceae, Aristolochiaceae, Asparagaceae, Asteraceae, Boraginaceae, Brassicaceae, Campanulaceae, Cannabaceae, Caprifoliaceae, Caryophyllaceae, Celastraceae, Convolvulaceae, Cornaceae, Crassulaceae, Cyperaceae, Elaeagnaceae, Ephedraceae, Euphorbiaceae, Fabaceae, Fagaceae, Frankeniaceae, Gentianaceae, Geraniaceae, Heliotropiaceae, Hypericaceae, Iridaceae, Juncaceae, Lamiaceae, Liliaceae, Linaceae, Lythraceae, Malvaceae, Moraceae, Oleaceae, Onagraceae, Orchidaceae, Orobanchaceae, Oxalidaceae, Papaveraceae, Plantaginaceae, Plumbaginaceae, Poaceae, Polygalaceae, Polygonaceae, Portulacaceae, Primulaceae, Ranunculaceae, Resedaceae, Rhamnaceae, Rosaceae, Rubiaceae, Rutaceae, Salicaceae, Sapindaceae, Scrophulariaceae, Solanaceae, Tamaricaceae, Thesiaceae, Thymelaeaceae, Ulmaceae, Urticaceae, Verbenaceae, Violaceae and Zygophyllaceae).

Now, after verification, it has proved necessary to remove *Astragalus* cfr. *varius* S.G.Gmel. and *Prangosodontalgica* (Pall.) Herrnst. & Heyn. In our publication and dataset is also one taxon distinguished in recent years - Limoniumtomentellum(Boiss.)Kuntzessp.alutaceum (Stev.), which is not yet in GBIF Backbone Taxonomy (GBIF Secretariat 2021).

### Taxa included

**Table:** 

Rank	Scientific Name	
kingdom	Plantae	
phylum	Tracheophyta	
class	Gnetopsida	
class	Liliopsida	
class	Magnoliopsida	
order	Apiales	
order	Asparagales	
order	Asterales	
order	Boraginales	
order	Brassicales	
order	Caryophyllales	
order	Celastrales	
order	Cornales	
order	Dipsacales	
order	Ephedrales	
order	Ericales	
order	Fabales	
order	Fagales	
order	Gentianales	
order	Geraniales	
order	Lamiales	
order	Liliales	
order	Malpighiales	
order	Malvales	
order	Myrtales	
order	Oxalidales	
order	Piperales	
order	Poales	
order	Ranunculales	
order	Rosales	
order	Santalales	
order	Sapindales	
order	Saxifragales	
order	Solanales	
order	Zygophyllales	
family	Adoxaceae	
family	Amaranthaceae	
family	Amaryllidaceae	
family	Anacardiaceae	
family	Apiaceae	
family	Apocynaceae	
family	Aristolochiaceae	
family	Asparagaceae	
family	Asteraceae	
family	Boraginaceae	
family	Brassicaceae	
family	Campanulaceae	
family	Cannabaceae	
family	Caprifoliaceae	
family	Caryophyllaceae	
family	Celastraceae	
family	Convolvulaceae	
family	Cornaceae	
family	Crassulaceae	
family	Cyperaceae	
family	Elaeagnaceae	
family	Ephedraceae	
family	Euphorbiaceae	
family	Fabaceae	
family	Fagaceae	
family	Frankeniaceae	
family	Gentianaceae	
family	Geraniaceae	
family	Heliotropiaceae	
family	Hypericaceae	
family	Iridaceae	
family	Juncaceae	
family	Lamiaceae	
family	Liliaceae	
family	Linaceae	
family	Lythraceae	
family	Malvaceae	
family	Moraceae	
family	Oleaceae	
family	Onagraceae	
family	Orchidaceae	
family	Orobanchaceae	
family	Oxalidaceae	
family	Papaveraceae	
family	Plantaginaceae	
family	Plumbaginaceae	
family	Poaceae	
family	Polygalaceae	
family	Polygonaceae	
family	Portulacaceae	
family	Primulaceae	
family	Ranunculaceae	
family	Resedaceae	
family	Rhamnaceae	
family	Rosaceae	
family	Rubiaceae	
family	Rutaceae	
family	Salicaceae	
family	Sapindaceae	
family	Scrophulariaceae	
family	Solanaceae	
family	Tamaricaceae	
family	Thesiaceae	
family	Thymelaeaceae	
family	Ulmaceae	
family	Urticaceae	
family	Verbenaceae	
family	Violaceae	
family	Zygophyllaceae	

## Temporal coverage

### Notes

2004 - 2009

## Collection data

### Collection name

Herbarium of Kherson State University (KHER), Herbarium of the Faculty of Biology of the University of Warsaw (WA), Herbarium in Paczovski Museum in Kherson.

### Specimen preservation method

driedAndPressed

## Usage licence

### Usage licence

Open Data Commons Attribution License

### IP rights notes

This work is licensed under a Creative Commons Attribution (CC-BY) 4.0 Licence.

## Data resources

### Data package title

Flora of kurgans in the "Wild Fields" (Ukraine)

### Resource link

https://www.gbif.org/dataset/59846cac-c4fd-4fde-bf45-5fe23d36f68f

### Alternative identifiers

https://ukraine.ipt.gbif.no/resource?r=ukrainian_kurgans

### Number of data sets

1

### Data set 1.

#### Data set name

Flora of kurgans in the "Wild Fields" (Ukraine).

#### Data format

Darwin Core

#### Download URL

https://www.gbif.org/dataset/59846cac-c4fd-4fde-bf45-5fe23d36f68f

#### Description

The dataset (Moysiyenko et al. 2022) includes a tabulation-delimited table with 29 fields in Darwin Core terms and contains 28,456 occurrences of vascular plants recorded on Ukrainian kurgans (barrows, ancient burial mounds). This is the first data collection from barrows in Ukraine. It contains information on the kurgan flora within four vegetation zones (the desert steppe, the grass steppe, the herb-rich grass steppe and the forest steppe).

The research (2004-2009) covered barrows on an area of 32000 km^2^. Of the approximately 450 kurgans visited, the ones with the best preserved vegetation cover were selected. For each of 106 investigated kurgans, floristic lists from five microhabitats were compiled (530 lists in total).

**Data set 1.** 

Column label	Column description
occurrenceID	An identifier of a particular occurrence, unique within this dataset. We used the species occurrence numbers (which indicates the specific climatic-vegetation zone, the particular kurgan and microhabitat and the frequency of species occurrence) (kurganplant.00001-kurganplant.28456).
scientificName	The original names as provided in publication (Sudnik-Wójcikowska, Moysiyenko et al. 2012), but corrected for spelling mistakes using GBIF Species Matching tool (with one exception – see Taxonomic coverage description).
organismQuantity	A number or enumeration value for the quantity of organisms. Estimated according to a 3-point scale: 1 – sporadic, 2 – infrequent, 3 – common.
organismQuantityType	The type of quantification system used for the quantity of organisms. We were used 3-point scale.
eventDate	The date-time or interval during which an Event occurred (2004-2009).
basisOfRecord	The method in which data were acquired (MaterialCitation).
geodeticDatum	The geodetic datum upon which the geographic coordinates are given (WGS84).
georeferencedBy	A person who determined the georeference.
georeferenceProtocol	A description or reference to the methods used to determine the spatial footprint, coordinates, and uncertainties (Manual with Google Earth GPS).
recordedBy	A person who responsible for recording the original Occurrence.
coordinateUncertaintyInMetres	The distance (in metres) from the given decimalLatitude and decimalLongitude describing the smallest circle containing the whole of the Location. Set from 7 m to 20 m for the coordinates georeferenced, based on description.
decimalLatitude	The geographic latitude in decimal degrees.
decimalLongitude	The geographic longitude in decimal degrees.
countryCode	The standard code for the country in which the Location occurs (UA).
country	The name of the country in which the Location occurs (Ukraine).
stateProvince	The name of the administrative region of Ukraine in which the Location occurs: Kherson, Mykolaiv, Poltava, Cherkasy, Kirovograd.
county	The full, unabbreviated name of the next smaller administrative region than stateProvince (districts).
habitat	A category or description of the habitat in which the Event occurred. Was divided into five microhabitats (T – the top of the barrow, Ss – the southern slope, Sn – the northern slope, Bs – the southern foot, Bn – the northern foot).
locality	The specific description of the place. The nearest village, the climatic-vegetation zone the number assigned to the specific kurgan
taxonRank	The taxonomic rank of the most specific name in the scientificName (genus, species, subspecies etc.).
kingdom	The full scientific name of the kingdom in which the taxon is classified. In our case, it is always Plantae.
phylum	The full scientific name of the phylum or division in which the taxon is classified. In our case, it is always Tracheophyta.
class	The full scientific name of the class in which the taxon is classified (Magnoliopsida, Liliopsida, Gnetopsida).
order	The full scientific name of the order in which the taxon is classified - Asterales, Lamiales, Caryophyllales etc. (Fig. 3; see also GBIF Database: Taxonomic distribution of occurrences).
family	The full scientific name of the family in which the taxon is classified. Fig. 3 (see also GBIF Database: Taxonomic distribution of occurrences).
verbatimIdentification	A brief phrase or a standard term ("aggr.", "cf.", "s.l.", "sp") to express the determiner's doubts about the Identification.
recordedByID	A list of the globally unique identifier for the people responsible for recording the original Occurrence.
identifiedByID	A list (concatenated and separated) of the globally unique identifier for the people responsible for assigning the Taxon to the subject.
associatedReferences	A list concatenated identifiers publication.

## Additional information

### Floristical richness and taxonomical value of kurgans in the “Wild Field”

**Figure 3.**
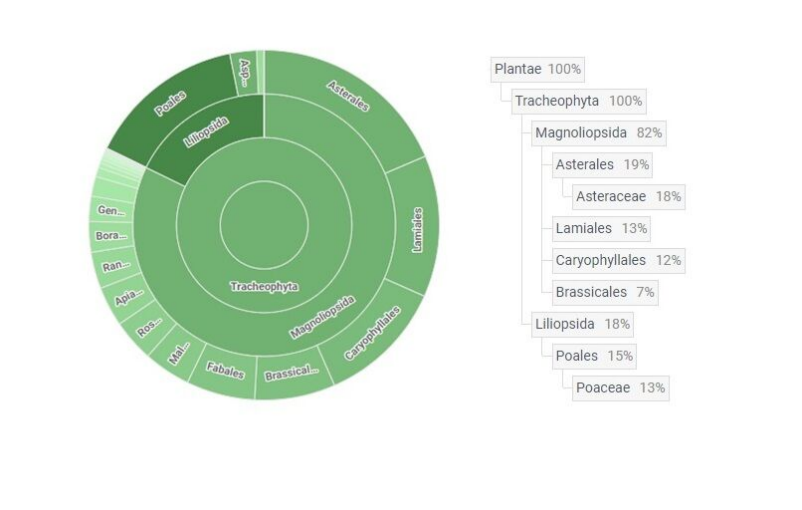
The taxonomic distribution of occurrences.

We identified 719 taxa of vascular plants on 106 kurgans, which make up 14.1% of the total flora of Ukraine (Mosyakin and Fedoronchuk 1999). Such a high number of species is not surprising, due to the large study area that has variable climatic conditions and different historical backgrounds. Most of the species belong to the class Magnoliopsida (Fig. 3). The most represented families in the kurgan flora are: Asteraceae, Poaceae and Fabaceae. These families are also well represented in the flora of Ukraine. The genera with the highest number of species identified on the kurgans were *Veronica* (18 species), *Trifolium* (12), *Astragalus* (10), *Euphorbia* (10), *Potentilla* (10) and *Centaurea* (9). The genera *Achillea*, *Artemisia*, *Carex*, *Galium*, *Vicia* and *Viola* were represented by eight species and *Allium* and *Salvia* by seven species.

### Floristic richness of microhabitats on kurgans

**Figure 4.**
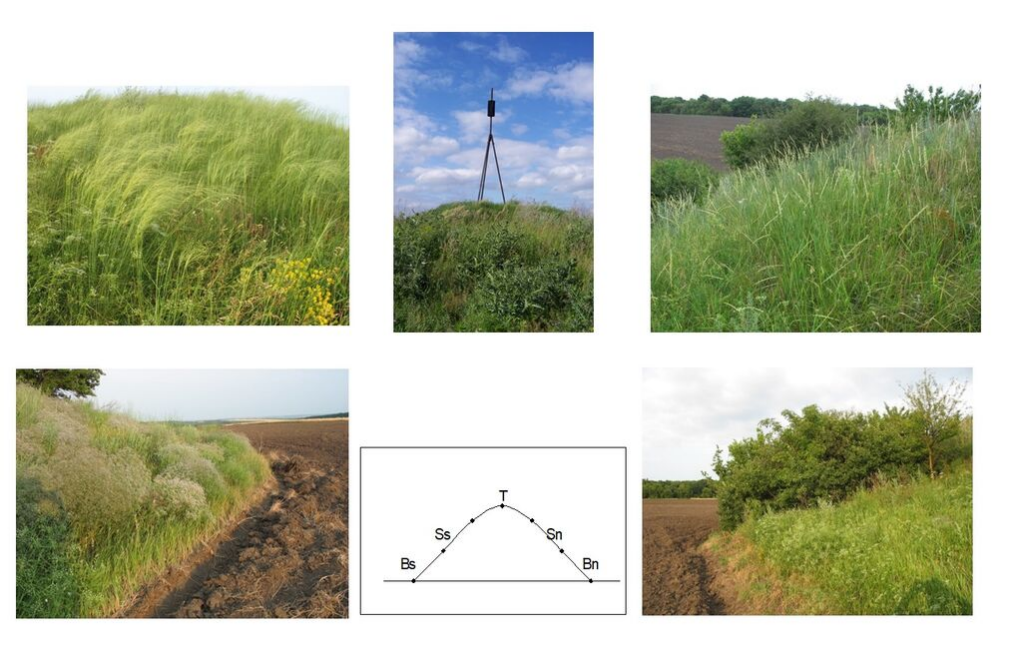
Microhabitat differentiation on kurgans: Bs – southern bottom; Ss – southern slope; T – top; Sn – northern slope; Bn – northern bottom.

The microhabitats within the kurgans vary with regard to different environmental conditions (Fig. 4, Table 2). Not only the southern slopes receive more solar radiation than the north facing ones, but also a vertical moisture gradient is observed. The rate of soil erosion varies depending on the site conditions (the top and base of the kurgan). All these factors imply differences in the composition of plant communities. For all 530 microhabitats at 106 kurgans, the lists of vascular plants were elaborated. On average, 54 species per microhabitat (min. – 13, max. – 117) were recorded. The floristic richness of microhabitats is growing from the top of kurgans to the bottom and from the southern exposition to the northern. As the average number of species of microhabitats increases, the following gradient occurs: T – Ss – Sn – Bs – Bn.

### Floristic richness of kurgans between zones

The long-term botanical studies conducted in the steppe and forest steppe zones of Ukraine (e.g. Lavrenko et al. (1991)) confirmed the high floristic diversity of these areas and showed that the species richness increase from south to north in the subsequent climatic-vegetation zones. This is associated with the gradually changing climatic conditions. In turn, the climate becomes milder towards the north (higher precipitation rates, lower summer temperatures).

Out of the total of 719 species recorded on the kurgans, 42% were represented in the desert steppe zone (located southwards) and 64% in the forest steppe zone (located northwards). The difference in the number of species between the kurgans in the four zones studied amounted to approximately 160 species. Our study confirmed (Table 3) that the total species richness on the kurgans was the lowest in the desert steppe zone (305 species), increased gradually in the grass steppe zone and the herb-rich grass steppe and was the highest in the forest steppe zone (460 species). In each zone, we examined almost the same number of barrows (25-29), which showed differences in the floristic richness, in accordance to the zone. The higher kurgan total flora diversity in the forest steppe could be also explained by a greater variety of biotopes on kurgans: from “dry steppe” on the tops and southern slopes, to more moist forest-like communities on the northern slopes and the bottom of the barrows. The anthropogenic influences and the occurrence of synanthropic species were probably also important.

On average, there are 107 species of vascular plants per kurgan. The floristic richness in the 106 examined barrows ranges from 48 to 189 species (Table 3). We used the mean number of species per kurgan in particular climate-vegetation zone as an indicator of floristic diversity. The mean number of species in the desert steppe was 82.3, in the grass steppe - 110 and in the herb-rich grass steppe - 125.5, respectively. The kurgans of the herb-rich grass steppe appeared to be the richest amongst kurgans examined in all other zones. Surprisingly, the mean number of species per kurgan in the forest steppe zone was 107.5. Despite the northernmost geographic location of the forest steppe zone, the particular barrows appeared to be less species-rich. This may be due to the fact that, in the forest steppe zone (at least in the area under investigation), it was much more difficult to find kurgans which would meet the set-up criteria. The kurgans were usually smaller (i.e. the mean height and diameter of the investigated forest steppe kurgans were 4.98 m and 54.1 m, respectively and, in the case of the herb-rich grass steppe zone, it was 5.7 m and 67.1 m, respectively) (Table 2). In addition, many of the kurgans were in poor condition. The worse condition of the barrows, at least locally, could be ascribed to the very early and still intensive agricultural practices in the forest steppe zone.

### Sozological value of the flora of Ukrainian kurgans

The kurgans are protected as archaeological monuments, but this is often not sufficient. The mounds are subjected to significant anthropogenic impacts. The main threats to the steppe flora on kurgans are: ploughing, archaeological excavations, illegal digging and looting, mechanical or agrichemical damages, afforestations, overgrazing, too frequent burning or mowing, littering, plant invasions from nearby agricultural landscapes etc. The kurgans have been influenced by some invasive alien species. This is evidenced by the presence of such species as: *Ailanthusltissima*, *Asclepiassyriaca*, *Eleagnusangustifolia* and *Grindeliasquarrosa*. Species have a tendency to inhabit the kurgans. Amongst these threats, it is especially unfortunate that the vegetation on the mounds is damaged by modern excavation technologies, during which the mounds are completely excavated and the vegetation is completely destroyed by archaeologists. A preventative action, from the point of view of habitat restoration, is the creation of protection zones around the mounds and creating of educational networks for understanding of value of kurgans. Moderate grazing, or haying, is also important to prevent "reserve succession". Reintroduction of vulnerable steppe species will also accelerate the natural process of restoration of typical vegetation. These preventative actions aiming at habitat restoration on kurgans, should concentrate on the creation of protection zones (buffer zones) around the mounds. The presence of buffer zones would prevent damaging the mounds by ploughing, as well as significantly reduce the ingress of pesticides. Kurgans, persisting within the intensively cultivated arable land of Ukraine, constitute the enclaves of natural steppe flora and an exceptional gene bank, which are representative for the particular climate-vegetation zones. Together with other fragments of natural or semi-natural steppe vegetation, they could play a significant role in the restoration of the European steppes, acting as “micro hotspots” and as donors of diaspores for the areas set free from intensive agricultural practices (Sudnik-Wójcikowska et al. 2012).

Amongst all recorded species, 69 (about 10%) were species of special concern, included in international and regional Red Lists (Sudnik-Wójcikowska et al. 2011). The sozophytes identified on the kurgans represented 26 families and 50 genera. The species represented the class Magnoliophyta (only one species belonged the class Pinophyta). Amongst the 69 taxa, 58 species occurred with frequency below 20% and four species with frequency between 20 and 40%. Two species were quite common: *Stipacapillata* (93%) and *Linariabiebersteinii* (74%). Rare species were present at all 106 kurgans. About 90% of the sozophytes recorded within kurgans had optimal conditions for growth on the slopes of the barrows. Only eight species (12%) were most frequently recorded at the base of kurgans (mesophilic or woody species). None of the species of special concern was associated with the top of a barrow – anthropophytes and hemi-apophytes clearly dominated in this type of microhabitat (Sudnik-Wójcikowska et al. 2011).

**Figure 5.**
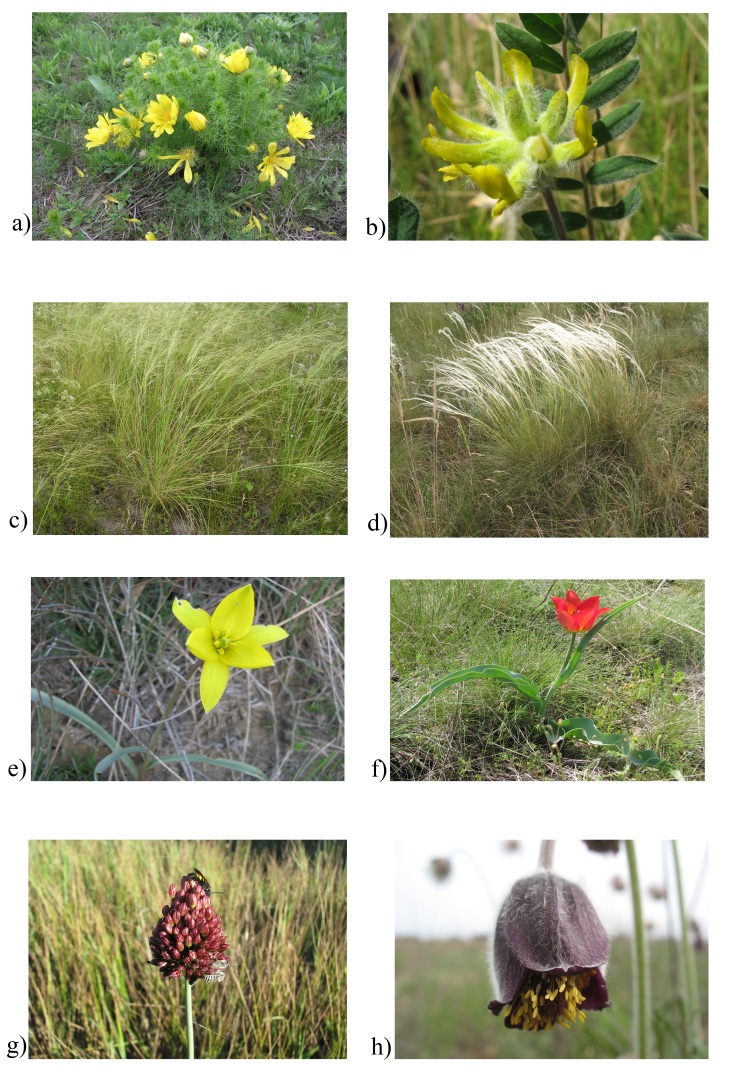
Rare species of the flora of investigated Ukrainian kurgans: **a)** *Adonisvernalis*; **b)** *Astragalusdasyanthus*; **c)** *Stipacapillata*; **d)** *Stipaucrainica*; **e)** *Tulipabiebersteinii* s.l.; **f)** *Tulipagesneriana*; **g)** *Alliumregelianum*; **h)** *Pulsatillapratensis* (photo Ivan Moysiyenko).

The dataset includes information about 1446 occurrences of rare species on kurgans. The occurrence of rare species on kurgans is growing from south to north and as follows: the desert steppe – 195 species, the grass steppe – 335 species, the herb-rich grass steppe – 418 species, the forest steppe – 498 species. The populations of these species are particularly vulnerable, because enclaves, such as kurgans, are usually small in size and isolated due to the huge fields around them. Anthropogenic factors (destruction of the base of the barrows by agricultural practices, the use of herbicides, mowing, grazing, trampling, illegal archaeological works etc.) also play a negative role. Populations of rare species on barrows are extremely sensitive and, therefore, require special measures to preserve them (Fig. 5).

## Figures and Tables

**Table 1.** Characteristics of the kurgans investigated in the steppe zones and forest steppe zone.

Characteristics of the investigated kurgans	Zones in which the kurgans were investigated			
	desert steppe (D)	grass steppe (P)	herb-rich grass steppe (R)	forest steppe (F)
Number of kurgans in particular zones	26	26	29	25
Mean height of the kurgans in particular zones (m) standard error	5.44 1.88	5.58 1.18	5.66 1.43	4.98 1.36
Minimum and maximum height of kurgans (m)	3.0-10.0	3.5-7.5	3.0-8.0	3.0-7.5
Mean diameter of kurgans in particular zones (m) standard error	56.44 18.44	62.31 14.16	67.07 16.61	54.12 11.26
Minimum and maximum diameter of kurgans (m)	32.5-90	35-80	40-100	38-80

**Table 2.** Floristic richness in microhabitats.

Microhabitats on kurgan	Minimum number of species in microhabitat per kurgan	Mean number of species in microhabitat per kurgan Standard error value	Maximum number of species in microhabitat per kurgan
Top (T)	15 / F21	33.8	70 / R3
Southern slope (Ss)	23 / F15	50.3	82 / P6
Northern slope (Sn)	27 / D26	56.8	104 / R3
Southern bottom (Bs)	14 / D26	61.0	117 / R17
Northern bottom (Bn)	13 / D26	66.5	112 / R2
Total for all microhabitats	13 / D26 / Bn	53.7	117 / R17 / Bs

**Table 3.** The basic parameters characterising the flora of kurgans in three types of steppe and forest steppe zones in Ukraine.

Characteristic of flora of investigated kurgans	Zone where kurgans were investigated			
	desert steppe (D)	grass steppe (P)	herb-rich grass steppe (R)	forest steppe (F)
Number of investigated kurgans	26	26	29	25
Total number of species	305	355	435	460
% of total kurgan flora (719 species)	42.3	49.2	60.3	63.8
Mean number of species per kurgan	82.3	110.0	125.5	107.5
Minimum and maximum number of species per kurgan	48-103	72-141	89-171	85-189
Number of kurgans with more than 100 species	5	20	25	16
Number of kurgans with more than 150 species	0	0	7	1
